# Crystal structure of 1,2-bis­(3,5-di­fluoro­phen­yl)ethane-1,2-dione

**DOI:** 10.1107/S2056989021005363

**Published:** 2021-06-08

**Authors:** Loren C. Brown, Gary J. Balaich

**Affiliations:** a Department of Chemistry & Chemistry Research Center, United States Air Force Academy, Colorado Springs, CO 80840, USA

**Keywords:** crystal structure, diketone, C—H⋯F inter­actions

## Abstract

The title compound, C_14_H_6_F_4_O_2_, crystallizes with half of a mol­ecule per asymmetric unit and exhibits bond lengths and angles typical of α-diketones. A network of C—H⋯F contacts and π–π stacking inter­actions is observed within the structure.

## Abstract 2

The title compound crystallizes with half of a mol­ecule per asymmetric unit and exhibits bond lengths and angles typical of α-diketones. A network of C—H⋯F contacts and π–π stacking inter­actions is observed within the structure.

## Chemical context   

Aryl diketones are a class of dicarbonyl compounds with a wide variety of uses in organic synthesis. The title α-diketone, 1,2-bis­(3,5-di­fluoro­phen­yl)ethane-1,2-dione, is used as a precursor in the production of hexa­benzocoronenes (Jones *et al.*, 2012 ▸). More recently, 1,2-bis­(3,5-di­fluoro­phen­yl)ethane-1,2-dione has been used in the synthesis of various polymers that have been studied for photovoltaics (Cai *et al.*, 2019 ▸) and for gas chromatography (GC) stationary phases (Liu *et al.*, 2019 ▸). Although the synthetic chemistry is known in the literature, to the best of our knowledge, structural data have not yet been published for the title compound. Herein we report the crystal structure of 1,2-bis­(3,5-di­fluoro­phen­yl)ethane-1,2-dione, isolated as a minor impurity in the synthesis of the related 1,4-di­aryl­ketone, 1-(3,5-di­fluoro­phen­yl)pentane-1,4-dione.
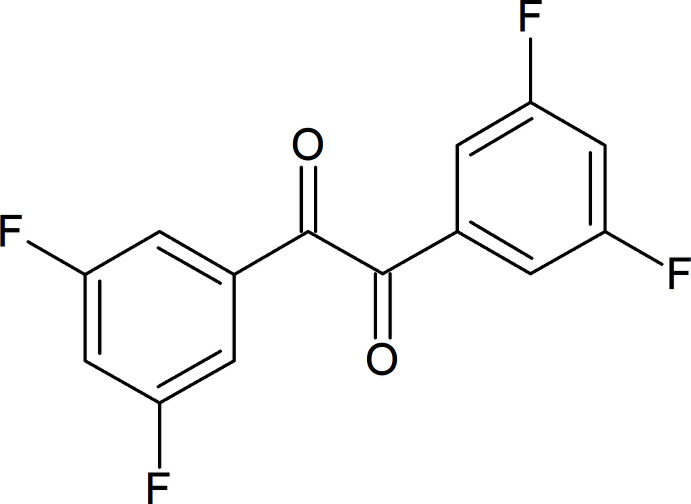


## Structural commentary   

**Figure 1:**
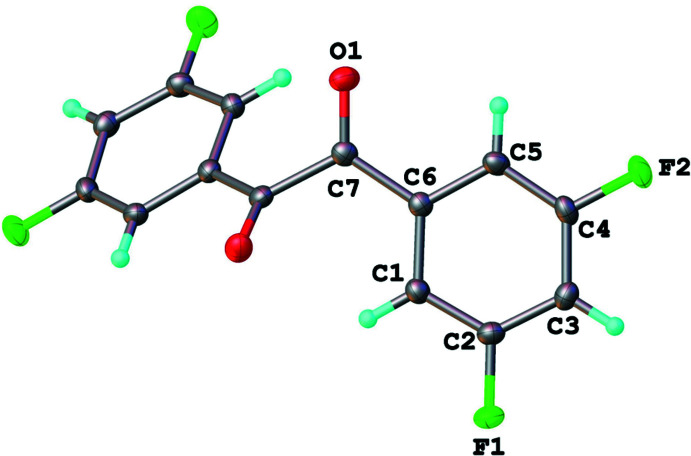
The mol­ecular structure of 1,2-bis­(3,5-di­fluoro­phen­yl)ethane-1,2-dione. Displacement ellipsoids are shown at the 50% probability level.

The title compound (Fig. 1 ▸) crystallized in the ortho­rhom­bic space group *Pbcn*. Benzil (1,2-di­phenyl­ethane-1,2-dione) and similar α,α-diketones crystallize in trigonal or monoclinic space groups, respectively (Charpe *et al.*, 2020 ▸; El Moncef *et al.*, 2010 ▸; Fun *et al.*, 2008 ▸). The title compound crystallizes with one half-mol­ecule per asymmetric unit (*Z*′ = 0.5), and exhibits the expected bond lengths and angles for α-diketone *sp* ^2^ hybridized atoms. Inter­estingly, the C5—C6—C7—O1 torsion angle [7.55 (19)°] of the title compound is larger compared to the same torsion angle in bis­(4-fluoro­phen­yl)ethane-1,2-dione [5.69 (18)°; Fun *et al.*, 2008 ▸] and benzil [3.80 (18)°; Charpe *et al.*, 2020 ▸], but smaller compared to 1,2-bis­(3-meth­oxy­phen­yl)ethane-1,2-dione [7.94 (15)°; Goossens *et al.*, 2005 ▸]. The dihedral angle between the two rings is 49.50 (6)° with a C6—C7—C7′—C6′torsion angle of 125.92 (5)°.

## Supra­molecular features   

**Figure 2:**
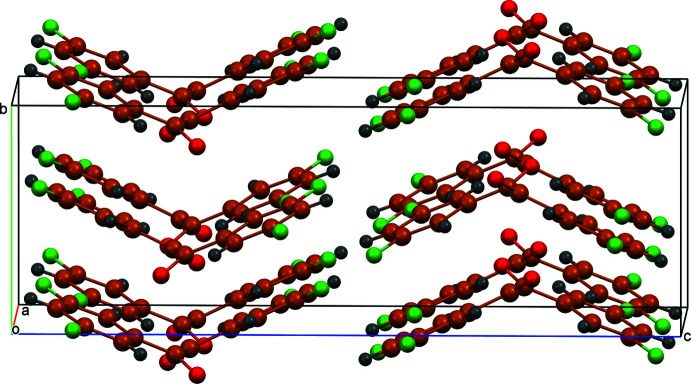
A packing view of 1,2-bis­(3,5-di­fluoro­phen­yl)ethane-1,2-dione.

**Figure 3:**
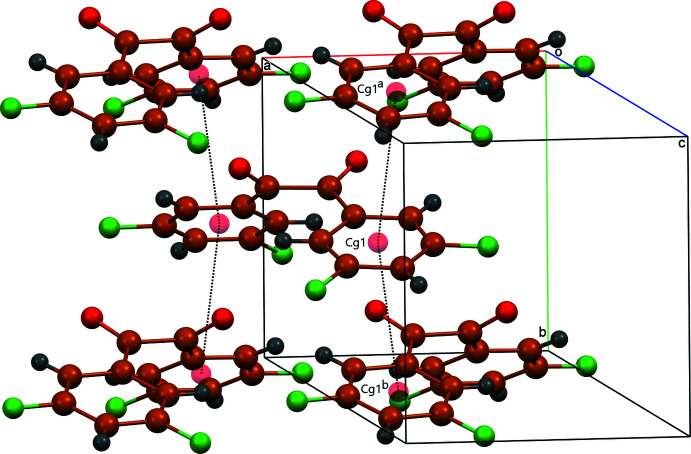
View of π–π stacking inter­actions in the title structure. Short inter­centroid separations are shown by dashed lines. *Cg*1 is the centroid of the C1–C6 ring.Symmetry codes: (*a*) 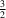 − *x*, −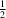 + *y*, *z*; (*b*) 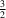 − *x*, 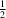 + *y*, *z*.

**Figure 4:**
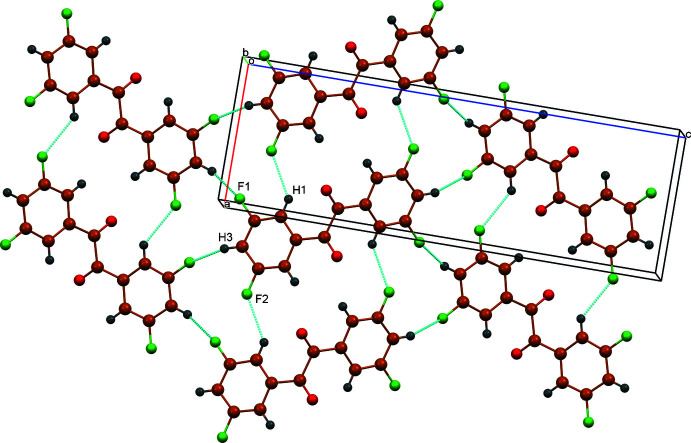
View of inter­molecular C—H⋯F inter­actions in the title structure (for details see Table 1 ▸).

A view of crystal packing of the title compound is presented in Fig. 2 ▸. The mol­ecules pack in a stacking pattern maximizing slipped π-π stacking inter­actions between planes of the di­fluoroaryl rings with an inter­centroid separation of 3.7317 (8) Å, thus forming layers parallel to the *bc* plane (Fig. 3 ▸). Similar π–π stacking inter­actions with comparable inter­centroid separations were observed in bis­(4-fluoro­phen­yl)ethane-1,2-dione [3.6416 (9) Å; Fun *et al.*, 2008 ▸] and benzil [3.7566 (17) Å; Charpe *et al.*, 2020 ▸]. As a result of the packing arrangement of bis­(3-meth­oxy­phen­yl)ethane-1,2-dione, no π–π stacking inter­actions were observed (Goossens *et al.*, 2005 ▸). The title compound packs in a way that allows close contacts between the fluorine atoms and hydrogen atoms of adjacent mol­ecules, leading to a network of C—H⋯F inter­actions (Table 1 ▸, Fig. 4 ▸) as well as fluorine inter­actions between neighboring mol­ecules [F1⋯F2(1 + *x*, *y*, *z*) = 2.9372 (16) Å, F1⋯F1(2 − *x*, 1 − *y*, 1 − *z*) = 2.8614 (16) Å]. A network of C—H⋯O inter­actions is also observed between the carbonyl oxygen and H5. This inter­action is significantly weaker for 1,2-bis­(3,5-di­fluoro­phen­yl)ethane-1,2-dione in comparison to benzil (O⋯H = 2.42 Å) and bis­(4-fluoro­phen­yl)ethane-1,2-dione (O⋯H = 2.40 Å). As a result, the π–π stacking and C—H⋯ F inter­actions play a vital role in how the compound packs within the crystal structure.

## Database survey   

A search of the Cambridge Structural Database (CSD, version of December 2019; Groom *et al.*, 2016 ▸) for aryl substituted α-diketones yielded 178 results. The bond lengths and angles in the title mol­ecule are consistent with α,α-diketones reported in the literature. The most closely related compound was compared to the title compound in the preceeding sections.

## Synthesis and crystallization   

Colorless crystals of the title compound suitable for single-crystal X-ray diffraction study were obtained by slow evaporation of a di­chloro­methane solution of the residue left after isolation of 1-(3,5-di­fluoro­phen­yl)pentane-1,4-dione.

## Refinement   

Crystal data, data collection and structure refinement details are summarized in Table 2 ▸. All H atoms were positioned geometrically (C—H = 0.93 Å) and refined as riding with *U*_iso_(H) = 1.2*U*_eq_(C).

## Supplementary Material

Crystal structure: contains datablock(s) I. DOI: 10.1107/S2056989021005363/yk2148sup1.cif

Structure factors: contains datablock(s) I. DOI: 10.1107/S2056989021005363/yk2148Isup2.hkl

Supporting information file. DOI: 10.1107/S2056989021005363/yk2148Isup3.cdx

Supporting information file. DOI: 10.1107/S2056989021005363/yk2148Isup4.cml

CCDC reference: 2085161

Additional supporting information:  crystallographic information; 3D view; checkCIF report

## Figures and Tables

**Table 1:** Hydrogen-bond geometry (Å, °)

*D*—H⋯*A*	*D*—H	H⋯*A*	*D*⋯*A*	*D*—H⋯*A*
C1—H1⋯F2^i^	0.93	2.48	3.2281 (16)	137
C3—H3⋯F1^ii^	0.93	2.46	3.3211 (15)	154
C5—H5⋯O1^iii^	0.93	2.65	3.517 (2)	156

Symmetry codes: (i) x+1, y, z; (ii) x-{\script{1\over 2}}, -y+{\script{3\over 2}}, -z+1; (iii) -x+1, y, -z+{\script{1\over 2}}.

**Table 2:** Experimental details

Crystal data	
Chemical formula	C_14_H_6_F_4_O_2_
*M*_r_	282.19
Crystal system, space group	Orthorhombic, *P* *b* *c* *n*
Temperature (K)	100
*a*, *b*, *c* (Å)	7.0588 (2), 7.4008 (2), 21.5265 (4)
*V* (Å^3^)	1124.56 (5)
*Z*	4
Radiation type	Mo *K*α
μ (mm^−1^)	0.16
Crystal size (mm)	0.30 × 0.14 × 0.10

Data collection	
Diffractometer	XtaLAB Synergy, Single source at offset/far, HyPix3000
Absorption correction	Gaussian (*CrysAlis PRO*; Rigaku OD, 2020 ▸)
*T*_min_, *T*_max_	0.679, 1.000
No. of measured, independent and observed [*I* > 2σ(*I*)] reflections	13347, 1202, 1014
*R*_int_	0.034
(sin θ/λ)_max_ (Å^−1^)	0.641

Refinement	
*R*[*F*^2^ > 2σ(*F* ^2^)], *wR*(*F* ^2^), *S*	0.032, 0.085, 1.03
No. of reflections	1202
No. of parameters	91
H-atom treatment	H-atom parameters constrained
Δρ_max_, Δρ_min_ (e Å^−3^)	0.21, −0.24

Computer programs: *CrysAlis PRO* (Rigaku OD, 2020 ▸), *SHELXT* (Sheldrick, 2015*a* ▸), *SHELXL* (Sheldrick, 2015*b* ▸), and *OLEX2* (Dolomanov *et al.*, 2009 ▸).

## supplementary crystallographic information

### Crystal data

**Table:**

C_14_H_6_F_4_O_2_	*D*_x_ = 1.667 Mg m^−^^3^
*M**_r_* = 282.19	Mo *K*α radiation, λ = 0.71073 Å
Orthorhombic, *P**b**c**n*	Cell parameters from 7273 reflections
*a* = 7.0588 (2) Å	θ = 1.9–27.0°
*b* = 7.4008 (2) Å	µ = 0.16 mm^−^^1^
*c* = 21.5265 (4) Å	*T* = 100 K
*V* = 1124.56 (5) Å^3^	Block, colourless
*Z* = 4	0.30 × 0.14 × 0.10 mm
*F*(000) = 568	

### Data collection

**Table:**

XtaLAB Synergy, Single source at offset/far, HyPix3000 diffractometer	1202 independent reflections
Radiation source: micro-focus sealed X-ray tube, PhotonJet (Mo) X-ray Source	1014 reflections with *I* > 2σ(*I*)
Mirror monochromator	*R*_int_ = 0.034
Detector resolution: 10.0000 pixels mm^-1^	θ_max_ = 27.1°, θ_min_ = 1.9°
ω scans	*h* = −8→9
Absorption correction: gaussian (CrysAlisPro; Rigaku OD, 2020)	*k* = −9→9
*T*_min_ = 0.679, *T*_max_ = 1.000	*l* = −27→26
13347 measured reflections	

### Refinement

**Table:**

Refinement on *F*^2^	Primary atom site location: dual
Least-squares matrix: full	Hydrogen site location: inferred from neighbouring sites
*R*[*F*^2^ > 2σ(*F*^2^)] = 0.032	H-atom parameters constrained
*wR*(*F*^2^) = 0.085	*w* = 1/[σ^2^(*F*_o_^2^) + (0.0435*P*)^2^ + 0.4818*P*] where *P* = (*F*_o_^2^ + 2*F*_c_^2^)/3
*S* = 1.03	(Δ/σ)_max_ < 0.001
1202 reflections	Δρ_max_ = 0.21 e Å^−^^3^
91 parameters	Δρ_min_ = −0.24 e Å^−^^3^
0 restraints	

### Special details

**Table:**

Geometry. All esds (except the esd in the dihedral angle between two l.s. planes) are estimated using the full covariance matrix. The cell esds are taken into account individually in the estimation of esds in distances, angles and torsion angles; correlations between esds in cell parameters are only used when they are defined by crystal symmetry. An approximate (isotropic) treatment of cell esds is used for estimating esds involving l.s. planes.

### Fractional atomic coordinates and isotropic or equivalent isotropic displacement parameters (Å^2^)

**Table:**

	*x*	*y*	*z*	*U*_iso_*/*U*_eq_	
F1	1.04484 (11)	0.64342 (12)	0.45793 (3)	0.0274 (2)	
F2	0.41188 (11)	0.52722 (13)	0.40758 (4)	0.0298 (2)	
O1	0.78193 (13)	0.28658 (13)	0.22568 (4)	0.0236 (3)	
C1	0.97690 (19)	0.50922 (17)	0.36170 (6)	0.0183 (3)	
H1	1.1050	0.5083	0.3516	0.022*	
C2	0.91452 (19)	0.57731 (18)	0.41774 (6)	0.0196 (3)	
C3	0.72718 (19)	0.58412 (18)	0.43505 (6)	0.0200 (3)	
H3	0.6896	0.6293	0.4734	0.024*	
C4	0.59783 (19)	0.51991 (19)	0.39216 (6)	0.0200 (3)	
C5	0.64773 (18)	0.44830 (18)	0.33558 (6)	0.0190 (3)	
H5	0.5563	0.4056	0.3081	0.023*	
C6	0.84090 (18)	0.44170 (17)	0.32055 (6)	0.0167 (3)	
C7	0.89509 (18)	0.36177 (17)	0.25965 (6)	0.0174 (3)	

### Atomic displacement parameters (Å^2^)

**Table:**

	*U*^11^	*U*^22^	*U*^33^	*U*^12^	*U*^13^	*U*^23^
F1	0.0235 (5)	0.0368 (5)	0.0218 (4)	−0.0053 (4)	−0.0033 (3)	−0.0094 (3)
F2	0.0151 (4)	0.0487 (6)	0.0254 (5)	0.0037 (4)	0.0047 (3)	−0.0003 (4)
O1	0.0235 (5)	0.0298 (6)	0.0176 (5)	−0.0056 (4)	−0.0014 (4)	−0.0014 (4)
C1	0.0160 (6)	0.0201 (7)	0.0188 (7)	−0.0005 (5)	0.0007 (5)	0.0009 (5)
C2	0.0203 (7)	0.0210 (7)	0.0174 (6)	−0.0013 (5)	−0.0030 (5)	−0.0009 (5)
C3	0.0232 (7)	0.0200 (7)	0.0168 (6)	0.0026 (6)	0.0025 (5)	−0.0003 (5)
C4	0.0142 (7)	0.0240 (7)	0.0220 (7)	0.0024 (5)	0.0028 (5)	0.0046 (5)
C5	0.0170 (6)	0.0230 (7)	0.0168 (6)	−0.0012 (5)	−0.0023 (5)	0.0029 (5)
C6	0.0169 (7)	0.0168 (7)	0.0162 (6)	0.0000 (5)	0.0005 (5)	0.0024 (5)
C7	0.0184 (7)	0.0178 (6)	0.0161 (6)	−0.0001 (5)	−0.0011 (5)	0.0036 (5)

### Geometric parameters (Å, º)

**Table:**

F1—C2	1.3543 (15)	C3—H3	0.9300
F2—C4	1.3550 (15)	C3—C4	1.3827 (19)
O1—C7	1.2176 (16)	C4—C5	1.3741 (19)
C1—H1	0.9300	C5—H5	0.9300
C1—C2	1.3796 (18)	C5—C6	1.4023 (18)
C1—C6	1.3985 (18)	C6—C7	1.4882 (17)
C2—C3	1.3748 (19)	C7—C7^i^	1.538 (3)
			
C2—C1—H1	121.1	C5—C4—C3	123.72 (13)
C2—C1—C6	117.73 (12)	C4—C5—H5	121.1
C6—C1—H1	121.1	C4—C5—C6	117.85 (12)
F1—C2—C1	118.28 (11)	C6—C5—H5	121.1
F1—C2—C3	117.84 (11)	C1—C6—C5	120.59 (12)
C3—C2—C1	123.87 (13)	C1—C6—C7	121.56 (12)
C2—C3—H3	121.9	C5—C6—C7	117.84 (11)
C2—C3—C4	116.21 (12)	O1—C7—C6	122.83 (12)
C4—C3—H3	121.9	O1—C7—C7^i^	117.99 (12)
F2—C4—C3	117.54 (12)	C6—C7—C7^i^	119.05 (12)
F2—C4—C5	118.74 (12)		

Symmetry code: (i) −*x*+2, *y*, −*z*+1/2.

### Hydrogen-bond geometry (Å, º)

**Table:**

*D*—H···*A*	*D*—H	H···*A*	*D*···*A*	*D*—H···*A*
C1—H1···F2^ii^	0.93	2.48	3.2281 (16)	137
C3—H3···F1^iii^	0.93	2.46	3.3211 (15)	154
C5—H5···O1^iv^	0.93	2.65	3.517 (2)	156

Symmetry codes: (ii) *x*+1, *y*, *z*; (iii) *x*−1/2, −*y*+3/2, −*z*+1; (iv) −*x*+1, *y*, −*z*+1/2.

### Selected bond distances (Å) and angles (°) for 1,2-bis(3,5-difluorophenyl)ethane-1,2-dione.

**Table:**

C1–C2	1.3796 (18)
C1–C6	1.3985 (18)
C2–C3	1.3748 (19)
C3–C4	1.3827 (19)
C4–C5	1.3741 (19)
C5–C6	1.4023 (18)
C6–C7	1.4882 (17)
C7–C7'	1.538 (3)
C7–O1	1.2176 (16)
F1–C2	1.3543 (15)
F2–C4	1.3550 (15)
	
C1–C6–C5	120.59 (12)
C1–C6–C7	121.56 (12)
C2–C1–C6	117.73 (12)
C2–C3–C4	116.21 (12)
C3–C2–C1	123.87 (13)
C4–C5–C6	117.85 (12)
C5–C4–C3	123.72 (13)
C5–C6–C7	117.84 (11)
C6–C7–C7'	119.05 (12)
O1–C7–C6	122.83 (12)
O1–C7–C7'	117.99 (12)
F1–C2–C1	118.28 (11)
F1–C2–C3	117.84 (11)
F2–C4–C3	117.54 (12)
F2–C4–C5	118.74 (12)

## References

[bb1] Cai, F., Li, L., Zhu, C., Li, J., Peng, H. & Zou, Y. (2019). *Chem. Phys. Lett.* **730**, 271–276.

[bb2] Charpe, V. P., Sagadevan, A. & Hwang, K. C. (2020). *Green Chem.* **22**, 4426–4432.

[bb3] Dolomanov, O. V., Bourhis, L. J., Gildea, R. J., Howard, J. A. K. & Puschmann, H. (2009). *J. Appl. Cryst.* **42**, 339–341.

[bb4] El Moncef, A., Cuquerella, M. C., Zaballos, E., Ramírez de Arellano, C., Ben-Tama, A., Stiriba, S. E. & Pérez-Prieto, J. (2010). *Chem. Commun.* **46**, 800–802. 10.1039/b915792a 20087525

[bb5] Fun, H.-K. & Kia, R. (2008). *Acta Cryst.* E**64**, o1617–o1618. 10.1107/S1600536808023350 PMC2962135 21203308

[bb6] Goossens, D. J., Welberry, T. R., Heerdegen, A. P. & Edwards, A. J. (2005). *Z. Kristallogr.* **220**, 1035–1042.

[bb7] Groom, C. R., Bruno, I. J., Lightfoot, M. P. & Ward, S. C. (2016). *Acta Cryst.* B**72**, 171–179. 10.1107/S2052520616003954 PMC4822653 27048719

[bb8] Jones, D. J., Purushothaman, B., Ji, S., Holmes, A. B. & Wong, W. W. H. (2012). *Chem. Commun.* **48**, 8066–8068. 10.1039/c2cc33892k 22767202

[bb9] Liu, J., Xu, L., Bai, J., Du, A. & Wu, B. (2019). *New J. Chem.* **43**, 8290–8298.

[bb10] Rigaku OD (2020). *CrysAlis PRO*, *CrysAlis CCD* and *CrysAlis RED*. Rigaku Oxford Diffraction, Yarnton, England.

[bb11] Sheldrick, G. M. (2015*a*). *Acta Cryst.* A**71**, 3–8.

[bb12] Sheldrick, G. M. (2015*b*). *Acta Cryst.* C**71**, 3–8.

